# A user-friendly web portal for T-Coffee on supercomputers

**DOI:** 10.1186/1471-2105-12-150

**Published:** 2011-05-12

**Authors:** Josep Rius, Fernando Cores, Francesc Solsona, Jano I van Hemert, Jos Koetsier, Cedric Notredame

**Affiliations:** 1 Department of Computer Science and Industrial Engineering, University of Lleida, C/Jaume II 69, E-25001 Lleida, Spain; 2 UK National e-Science Centre, University of Edinburgh, 10 Crichton Street, EH8 9AB, Edinburgh, UK; 3 Centre For Genomic Regulation (Pompeu Fabra University), C/Doctor Aiguader 88, 08003 Barcelona, Spain

## Abstract

**Background:**

Parallel T-Coffee (PTC) was the first parallel implementation of the T-Coffee multiple sequence alignment tool. It is based on MPI and RMA mechanisms. Its purpose is to reduce the execution time of the large-scale sequence alignments. It can be run on distributed memory clusters allowing users to align data sets consisting of hundreds of proteins within a reasonable time. However, most of the potential users of this tool are not familiar with the use of grids or supercomputers.

**Results:**

In this paper we show how PTC can be easily deployed and controlled on a super computer architecture using a web portal developed using Rapid. Rapid is a tool for efficiently generating standardized portlets for a wide range of applications and the approach described here is generic enough to be applied to other applications, or to deploy PTC on different HPC environments.

**Conclusions:**

The PTC portal allows users to upload a large number of sequences to be aligned by the parallel version of TC that cannot be aligned by a single machine due to memory and execution time constraints. The web portal provides a user-friendly solution.

## Background

Nowadays, researchers in the field of genomics increasingly require greater computational resources to perform their experiments. Until recently, typical biological datasets consisted of less than a 100 sequences. A well-endowed desktop computer was therefore sufficient to produce a satisfactory multiple sequence alignment with most software. The situation has now dramatically changed with recent progress in large-scale genomics resulting in the availability of biologically meaningful datasets comprising thousands of sequences. One of the most immediate consequence is the need of alignment tools designed for large-scale analysis that are able to process thousands, or even tens of thousands, of sequences. The computational requirements of these problems consists of months of computer time and terabytes of memory, which in turn requires High-Performance Computing (HPC) systems with thousands of processors, also known as supercomputers. Unfortunately, most supercomputers still provide only very basic user interfaces based on terminals used over several decades. We address this issue by introducing a user-friendly portal that provides controlled access in a versatile way to an accurate multiple sequence alignment tool--Parallel T-Coffee [1]--that runs efficiently on the required computing resources (supercomputer or Grid). Thereby we allow large-scale alignments. The web portal was developed with Rapid [2]--a specialised tool that provides a cost-effective and efficient way of designing and delivering portal interfaces to applications requiring remote computational resources. The aim of Rapid is to make the use of remote computing applications as easy as booking a flight or purchasing a book on the Internet.

### Parallel T-Coffee

Parallel T-Coffee (PTC) [1] is a parallel version of the T-Coffee (TC) multiple sequence alignment (MSA) program [3]. MSA programs are meant to align a set of homologous sequences previously gathered using homology search programs such as Blast. The main characteristic of TC is that it combines the consistency-based scoring function Coffee [4] with the progressive alignment algorithm. The strategy developed for TC now forms the basis of many new generation aligners [5]. The main advantage of consistency is to make it less likely for early stage errors to compromise the computation of an accurate MSA. In TC, consistency is estimated using a library made of pairwise global and local alignments whose information content is combined within the final MSA. Although it results in more accurate alignments, the use of consistency also comes at an increased computational cost. So far this limitation has hampered the use of consistency based method in the context of large scale analysis. Taking this into account, PTC allows to compute alignments of more than hundreds of sequences, far beyond the capability of the sequential version of TC. PTC is implemented on version 3.79 of TC and supports most of the options provided by this package. The PTC implementation is based on distributed-memory architectures (for example a supercomputer or a cluster of computers), using a message passing paradigm and one-sided communication primitives [6] to exchange information among the different tasks. The main stages of T-Coffee - library generation and progressive alignment - have been parallelized in PTC in order to be executed in different compute-nodes.

New versions of T-Coffee package [7] also allow parallel executions. However, its approach is different, it is based on shared-memory architectures, like multi-core or multi-processor ones. Nowadays, due to the fact that distributed-memory systems do not have the memory size limitation imposed by the shared-memory architectures, it can be said that PTC has an increased scalability. Therefore, PTC can take advantage of the aggregate resources (CPU and memory) of thousands of processors/cores interconnected by high-speed networks. The only inconvenient of this approach is that message-passing paradigm is much more complex than shared-memory one, requiring more development time.

### Rapid

Rapid [2] is a cost-effective and efficient way of designing and delivering portal interfaces to tasks that require remote compute resources. The philosophy of Rapid is to deliver customized graphical user interfaces that enable domain specialists to achieve their tasks. These tasks make use of domain-specified applications that run on remote computational resources; a requirement which is satisfied by translating the task into one or several computational jobs to be performed on Grids, Cloud Computing infrastructures, or supercomputers. Customized interfaces allow tasks to be performed without referring to terminology about the underlying computational infrastructure. Moreover, a customised web portal can make the application itself easier to use by only exposing the functionality required in the task and by adding additional checks to validate input.

## Implementation

**Figure 1:**
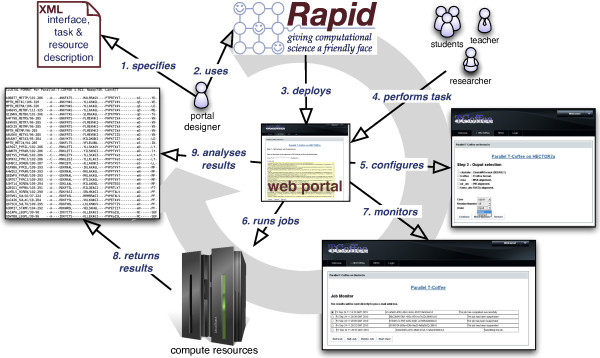
**Rapid diagram**. Process of creating and using Parallel T-Coffee portlets via Rapid.

We have chosen Rapid to develop the web service because it is an easy and fast way to develop HPC portals. The full process for creating the portlet using this tool is shown in Figure 1. In the first phase a specification file using *Extensible Markup Language *(XML) was created. This file describes the interface, task and resource description and is used by Rapid to generate the JSP files containing all the portlet. In other words, from this one file the whole user interface is then generated.

Due to XML's limitations, Rapid is extended with and interfaced for Jython plugins that allows the web designers to add more complex logic and dynamic user interfaces. This functionality has been very useful in the development process of the PTC portal allowing us to create a more friendly and dynamic framework. Rapid also generates automatically a Java WAR file used to deploy the portlet in a large variety of portal containers. This file contains all the necessary data and can be generated with the specific requirements of different deployment descriptors for each portal vendor, thus making the deployment process as generic as possible. In this work, we use LifeRay as the portal container to deploy our PTC portal.

As any standard web interface, the resulting portal directs the user through the submission process of the sequences to be aligned:

1. *Basic submission: *Users can easily upload their set of sequences and apply directly for the alignment without care about several execution options offered by PTC.

2. *Advanced options: *Advanced users can opt for a more personalized alignment by choosing between a multitude of options. Actually, the most important options of PTC have been taking into account in the design phase of the portal.

3. *Monitor: *The job monitor page displays information about submitted data sets, the date of the submission and their state.

4. *Results: *Finally, the results are sent directly by e-mail to the user, so one can exit the web portal once the job has been submitted.

## Results and Discussion

The PTC portal allows users to upload a large number of sequences to be aligned by the parallel version of TC that cannot be aligned by a single machine due to memory and execution time constraints. The main interest here is not so much the portal, but rather the methodology used to generate it, using a collection of tools that allow the seamless integration of a supercomputer environment with an optimised parallel application. Yet, the resulting portal also has a concrete application that allows users to compute T-Coffee alignments on large datasets in a time comparable to that measured on other servers running faster but less accurate packages. The results showed in [8] have demonstrated that Parallel T-Coffee can decrease the time required for the alignment of the PFAM database by one order of magnitude with respect to the serial version. With this speed-up, Parallel T-Coffee can reach execution times similar to those obtained with ProbCons or Dialign, taking into consideration the experimentation performed in [9].

**Figure 2:**
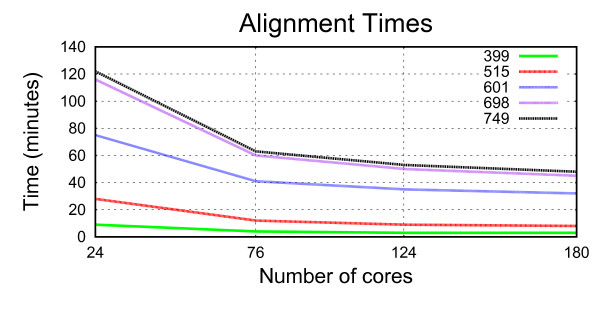
**Alignment Times**. Graph representing the execution time of each alignment in minutes depending on the number of processors and the number of sequences.

For the experimental results, we have used different protein families from PFAM database [10] with different number of sequences (399, 515, 601, 698 and 749) and different average sequences lengths (119, 194, 164, 164 and 164, respectively). Table 1 shows the time that PTC takes to align different numbers of sequences depending on the number of cores used in each test. The times have been obtained using the HECToR supercomputer and using the PTC portal for launching the jobs. The complexity of T-Coffee algorithm is *O*(*N*^2^*L*^2^) [1], with *N *being the number of sequences to be aligned and *L *the average sequence length. This complexity means that the execution time increases quadratically with the number and the length of the sequences. Therefore, when the alignment complexity increases, the use of PTC is necessary as it can obtain larger speed-ups. However, it can be seen in Figure 2 that the speed-up is limited because the latter stages of the alignment have poor parallelism due to their tree structure.

**Table 1:** Alignment times

*^Cores^*/*_Seq_*	399	515	601	698	749
24	9'	28'	1 h 15'	1 h 56'	2 h 2'
76	4'	12'	41'	1 h	1 h 3'
124	3'	9'	35'	50'	53'
180	3'	8'	32'	45'	48'

**Figure 3:**
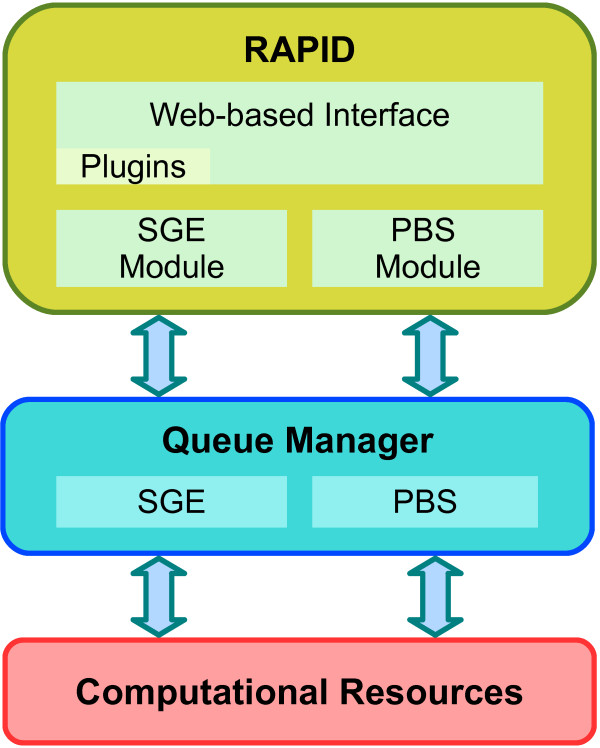
**Portal architecture**. Multi-layer architecture of Parallel T-Coffee portal.

The PTC web service that we present in this paper is currently suited to run on the HECToR supercomputer located at the University of Edinburgh. It can be downloaded and easily adapted to any other cluster or supercomputer that makes use of SGE, PBS or Condor as the queue manager. Figure 3 shows the layer architecture of how the PTC web-based interface works over different kinds of supercomputers. As can be seen, the communication with the queue manager is implemented into separate modules. That feature allows developers to adapt in an easy way the PTC portal on clusters running with different queue manager systems. Furthermore, this modular implementation also facilitates the adaptation process of the portal into other clusters since the administrators only need to change some properties of these modules without the necessity to know anything about the rest of the code.

## Conclusions

We introduced a web portal created with Rapid that improves the use of PTC on supercomputers by making the user interface intuitive. The idea is to provide users who want to align a set of sequence using a high accuracy method, such as T-Coffee, an alternative to the TC web service without the single-machine memory limit. Furthermore, we have developed this portal taking into account mainstream job queue managers, i.e. SGE, PBS and Condor. This allows anyone to quickly adapt our web interface to be used on other supercomputers, compute clusters or Grids.

## Availability and requirements

In this section, the main availability and requirements details are provided.

• Project name: Parallel T-Coffee Portal

• Project home page: http://gcd.udl.cat/ptc

• Operating system: Platform independent

• Programming language: Xml + Jython

• Other requirements: Tomcat 4.0 or higher and a portal container e.g. LifeRay

• License: Gnu General Public Licence

• Any restrictions to use by non-academics: none declared

## List of abbreviations

TC: T-Coffee; PTC: Parallel T-Coffee; MSA: Multiple Sequences alignment; HPC: High Performance Computing; MPI: Message Passing Interface; RMA: Remote Memory Access; XML: eXtensible Markup Language; WAR: Web Application aRchive; JSP: JavaServer Pages; SGE: Sun Grid Engine.

## Authors' contributions

JK and JvH developed Rapid, which was used for the Parallel T-Coffee portal and JR was responsible for the implementation process. The validation test of the web-based interface were performed by FC and FS The sequence alignments were done by CN using the PTC portlet. The paper was written by JR and proofread and edited by the co-authors. The supervision of JvH and CN from the conception of the project was essential to its success. All authors read and approved the final manuscript.

## Authors information

JR received his BS and MS in computer science from Universitat de Lleida (UdL) in 2006 and 2008 respectively. Currently hi is a PhD student in the same university and his research interests are high-performance computing, P2P systems and parallel simulation.

FC received his BS and MS from 1997 and 2000, respectively, and a PdD in distributed multimedia systems from the Universitat Autonoma of Barcelona, Spain, in 2004. He is an assistant professor in the Computer Science Department of the University of Lleida, Spain. His research interests include distributed multimedia systems, multimedia and high-performance P2P systems and parallel simulation.

FS received the B.S., M.S. and Ph.D. degrees in computer science from the Universitat Autònoma de Barcelona, Spain, in 1991, 1994 and 2002 respectively. Currently, he is an associate professor in the Department of Computer Science at the University of Lleida (Spain). His research interest include are distributed processing and HPC.

JvH has a PhD in Mathematics and Physical Sciences from the Leiden University, The Netherlands (2002). He is a project manager at Optos--an innovative retinal imaging company with a vision to be recognised as the leading provider of retinal diagnostics. Since 2010, he is a Honorary Fellow of the University of Edinburgh.

JK is a research associate at the UK's National e-Science Centre of the University of Edinburgh. His research interests are grid computing. He has an MSc from University of Twente in the Netherlands and a PhD from the University of the West of Scotland (formerly the University Paisley) in the UK.

CN is a group leader in the Bioinformatics and Genomics programme of the CRG. He was trained as a bioinformaticist in the lab of Des Higgins at EMBL (Heidelberg) and later at the EBI-EMBL (Cambridge). He was awarded his PhD in 1997 and then obtained a junior professor position in Marseille University (1999) and in Lausanne University (2000). In 2002 he obtained a junior CNRS scientist position. After 5 years in the lab of Jean Michel Claverie (IGS) he is currently on leave at the CRG (Barcelona) where he is a senior group leader, heading the laboratory of Comparative Bioinformatics in the Bioinformatics and genomics department. His work is focused on the development of multiple sequence alignment methods and the comparison of protein sequences and structures. He has developed and maintained the T-Coffee Multiple sequence alignment Package, one of the most accurate of its kind that has so far received close to 1000 citations. Between 2001 and 2006, he was a consultant for the pharmaceutical industry (Aventis and Sanofi Aventis) providing expertise on the analysis of the human kinome through multiple sequence comparisons.
